# The Importance of Critically Examining the Level of Propositions When Evaluating Forensic DNA Results

**DOI:** 10.3389/fgene.2016.00008

**Published:** 2016-02-08

**Authors:** Alex Biedermann, Tacha Hicks

**Affiliations:** Faculty of Law, Criminal Justice and Public Administration, School of Criminal Justice, University of LausanneLausanne, Switzerland

**Keywords:** level of propositions, evaluative reporting, European guidelines

## Introduction

The proposal of a discussion about the use of software to help assign likelihood ratios for forensic DNA profiling results, and the use of their output in the legal process, is both timely and important (see also related contributions elsewhere in *Frontiers*, e.g., Biedermann et al., 2014). Ever since their introduction in forensic science, DNA profiling analyses have been accompanied with the results of calculations of various sorts. Their scope is well illustrated and documented in several reference monographs (e.g., Evett and Weir, 1998; Buckleton et al., 2005; Balding and Steele, 2015). This solid body of scholarly research and established practice has contributed to the widely held view among scientists and recipients of expert information that eliciting the probative strength of forensic DNA profiling results is *per se* a numerical task.

In this commentary, we intend—in a first part—to make the point that although calculations are, by virtue, an integral part of the quantification of probative strength, it is equally important at the outset to be clear about the question “Why are we doing a calculation?” (Buckleton et al., 2005, p. 151). We will argue that this is not a question that statistics can answer. Stated otherwise, we will contend that, as much it is important to be clear in any instance about what a particular computation exactly purports to do, it is essential to define the questions that are of interest in a particular case at hand. In a second part, we will emphasize on the extent to which, why and how recently issued guidelines (e.g., ENFSI, 2015) encourage such thinking about cases prior to conducting calculations, if any.

## Questioning default calculations

Experience demonstrates that many scientists working in operational laboratories decide on the use of particular computational procedures—often provided by ready-to-use software packages—based on the mere availability of those procedures at their workplace. This amounts to a convenience choice, but what is more is that proceeding in this way is considered the best one could do. This view may be reinforced if the software is based on Bayesian principles, because procedures that belong to this class of inferential methods are referred to as the most inferentially sound. But the sole fact that a procedure relies on Bayesian principles does not make it *per se* pertinent for the case at hand. As noted by Lindley (2004, p. 74), “[t]he main danger is that they [Bayesian methods; *added by the authors*] will be used automatically. (…) You must think about the real quantities involved, like temperature or blood pressure, and not about symbols that represent them. This distinction between the thinking you and the unthinking, calculating personal computer is essential.” This danger also exists in the context of interpreting and reporting forensic DNA results. Indeed, most of the commonly available computational procedures^1^ lead to expressions of probative strength to help discriminate between so-called sub-source level propositions [e.g., “the person of interest (POI) is the source of the recovered DNA” vs. “an unknown person is the source of the recovered DNA”]. But, in many practical cases, the real question goes beyond this level, e.g., how the detected DNA got where it was found (Evett et al., 2002; Taroni et al., 2013), that is so-called activity level propositions. Cases of alleged rape where the competing versions only differ with respect to the activities that led to the trace illustrate this. This is of course not a critique of models being Bayesian in nature, but of the kind of questions to which some of these models are tailored.

Skeptics may invoke that none of the above problems are novel. But why then practice by and large remains unchanged? While some scientists openly acknowledge that expressions of probative strength of DNA considering sub-source level propositions may indeed be insufficient for the needs, some hold that it is for the Court to decide on that matter. We perfectly agree with this stance, of course, because whatever the level of the propositions, it is for the Court to decide on the probability of the propositions. Notwithstanding, scientists can add considerable value by assessing their results *given* activity level propositions.

Yet others contend that one can leave this debate until the Courtroom. However, this may raise issues from a quality management point of view, and render the situation very uncomfortable for the witness, because of the inevitable difficulty of the task. The challenge is real for a variety of case scenarios, in particular where only low quantities of DNA are detected and/or when POIs do not deny that the recovered DNA is theirs. We seriously doubt that members of the judiciary are able to properly appreciate the extent to which one can expect to obtain a low quantity of DNA, recovered at a certain position on the crime scene, the victim, or a POI, given one activity as compared to another activity. We would not recommend either doing this evaluation on the stand. This is because such assessments are very challenging even for experts, and require *scientific knowledge* about many factors, such as transfer, persistence, and the capacity of a given donor to shed detectable quantities of DNA^2^. Let us emphasize again that the question of whether the detected DNA is that of the POI may be entirely uncontested (and thus there would be no need for a likelihood ratio given sub-source propositions as there is no uncertainty about sub-source). What is really of interest is to assess the probability of observing such a result for a DNA trace, that is a trace found in a particular position, in a given quantity and leading to a profile of the observed quality given the alleged activities and given relevant information such as the time lapse between collection of trace material and the commission of the crime, environmental factors to which the trace was exposed (e.g., temperature, humidity) etc. Such assessments are highly case dependent, which calls for the generation of more research with experiments under controlled conditions, that can help build a community-wide knowledge base (Evett, 2015)^3^. To further emphasize the need for considering observations given activity level propositions, note again that the result which is to be assessed is *not only* the rarity of genetic features, but also extends to the very fact of finding, at a given position, a detectable quantity of DNA (Evett and Weir, 1998), which may be nil. Sub-source level propositions cannot deal with results that did not yield a DNA profile.

The mismatch between default evaluations given sub-source level propositions and the decision makers' interest in activity level propositions is a cause of concern because the strength of the observations in the former case can be radically different from that of the latter, so that inappropriate conclusions can result if the two are taken to be equivalent. We have seen this happen in cases where scientists report likelihood ratios in the order of >10^20^ with propositions at sub-source level when in fact the real issue was one of activities and where the strength of the findings, given the conditioning information of the case at hand, was way more moderate^4^.

## Current recommendations

The above discussion is not intended to suggest that evaluation given (sub)-source level propositions is useless or detrimental in principle^5^. The point we seek to make is that it is crucial to assess the needs of the recipient of expert information prior to choosing a computational procedure. This seems like an obvious and moderate requirement, yet experience shows that often it is given little attention in practice. Recent works by forensic scientists from across Europe, published in the form of a guideline (ENFSI, 2015), seek both to strengthen awareness of this issue and help scientists and recipients of expert information proceed in a more sensible way. For example, in its Guidance Note 2 on propositions, the document specifies: “Source level propositions are adequate in cases where there is no risk that the court will misinterpret them in the context of the alleged activities in the case” (ENFSI, 2015, p. 12). To illustrate this idea, the following example is given: “A large fresh bloodstain is recovered at the point of entry at a burglary scene and delivered to the laboratory for DNA analysis. Combination of a presumptive test and appearance allows the scientist to safely assume that the stain is blood. A suspect says that he has never been in the premises. The set of propositions can be (1) the bloodstain came from the defendant and (2) the bloodstain came from another unknown individual” (ENFSI, 2015, p. 12). In this example, source level propositions are *not* problematic because no expert knowledge is required regarding phenomena such as transfer and persistence, as well as background levels of DNA. Such factors do not impact, in this kind of circumstances, on the understanding of scientific findings relative to the alleged activities. In particular, it is not doubted that the bloodstain results from the act of breaking in. This example also illustrates that there is more to the collected trace than the DNA profile: there are aspects such as the freshness of the stain, the quantity of material and the position where the trace was found. In turn, it is clear that specialized knowledge regarding transfer, persistence and background *would matter* in the above scenario *if* DNA had been detected in low quantities, rather than from a rich bloodstain.

The above understanding has far reaching implications: the level of propositions depends on the factors and observations on which forensic scientists have expert knowledge. It is their duty to evaluate all their results so that the Court is not deprived of information that is necessary for a balanced view. For example, the ENFSI guideline explicitly advises against the changing of propositions from activity to (sub-) source level when relevant expert knowledge is not available: “In fact, the choice between (sub-) source and activity should not be influenced by the availability of data or expert knowledge but solely from the consideration of factors such as transfer, persistence, and background levels that could crucially affect the strength of the findings within the context of the case circumstances.” (ENFSI, 2015, p. 13).

We acknowledge, from personal experience, that the implementation of the above perspective is challenging. It may be even more so in systems exposed to commercialisation where forensic providers that conduct DNA profiling analyses operate more and more separated from those entities that collected trace material at the crime scene (Jackson, 2013). Further obstacles may be operational constraints such as time and costs, because evaluation given activity level propositions does not rely on default computations, but generally requires a case-based approach. Regarding the latter point, some scientists deplore a lack of formulaic developments for evaluation given propositions at higher hierarchical levels. But this critique does fall short of the current state of developments. Formal likelihood ratio approaches exist (e.g., Evett, 1984; Evett and Weir, 1998), used also for other transfer materials (e.g., glass; Curran et al., 2000), and there are reports that demonstrate the relevance and practical feasibility (e.g., McKenna, 2013). Yet, other developments allow one to account for uncertainty about the relevance of the recovered material and the possibility that material was left for innocent reasons (e.g., Evett, 1993; Evett et al., 2002).

The role of statistics in evaluating DNA profiling evidence has always been important, but we now must realize that, increasingly often, the traditional perspective of sub-source level propositions, and the main focus on the rarity of the corresponding features (i.e., the so-called conditional genotype probability), may represent only a first step of the evaluative process. This does not make these evaluation approaches wrong, only less comprehensive. The fact is that the extrinsic characteristics of the trace material (i.e., low quantities of DNA) and the propositions of interest have changed, and it is important to realize that this represents the relevant starting point. This recognition of the needs cannot be answered by statistics, only the evaluative procedures that need to be built once the needs are properly elicited. The importance of statistics in this endeavor remains unaffected, and stands as noted by Lindley (2000, p. 38): “(…) the first task of a statistician is to develop a (probability) model to embrace the clients' interests' and uncertainties. It will include the data and any parameters that are judged necessary. Once accomplished, the mechanics of the calculus take over and the required inference is made.”

## Author contributions

Both authors have made substantial, direct, and intellectual contribution to the work, and approved it for publication.

### Conflict of interest statement

The authors declare that the research was conducted in the absence of any commercial or financial relationships that could be construed as a potential conflict of interest.

## Footnotes

^1^The scope of these procedures is large and includes topics such as complex modeling of products (e.g., stutters) of the PCR amplification of STRs (e.g., Bright et al., 2014; Gittelson et al., 2014) and the study of the sensitivity of expressions of probative value due to the use of particular statistical techniques (e.g., MCMC techniques, see for example Bright et al., 2015).

^2^On this topic, see for example http://www.telegraph.co.uk/news/science/9115916/The-case-against-DNA.html.

^3^For an example in other transfer traces, see Buckleton et al. (1989).

^4^Another issue, not pursued here, is whether likelihood ratios exceeding one over the earth population, and multiples of that, are reasonable. There is much argument to say they are not (e.g., Thompson et al., 2003; Hopwood et al., 2012).

^5^In fact, the strength of the DNA correspondence is so high that this will lead to situations where the source of the DNA will be admitted (leaving no uncertainty on the source of the DNA). This, then moves the issue to the activities.
